# Spatial transcriptomic profiling of porcine tissue microarray detecting subclinical circovirus infection

**DOI:** 10.3389/fvets.2026.1900962

**Published:** 2026-09-17

**Authors:** Wooseok Kim, Sunmin Song, Hyeonjeong Cho, Jong-Eun Park, Taehwan Oh

**Affiliations:** 1 Graduate School of Medical Science and Engineering, Korea Advanced Institute of Science and Technology (KAIST), Daejeon, Republic of Korea; 2 Department of Microbiology, College of Medicine, Dankook University, Cheonan, Republic of Korea; 3 Systems Virology and Spatial Immunomics Group, Center for Virome and Applied Platform, Korea Virus Research Institute, Institute for Basic Science, Daejeon, Republic of Korea

**Keywords:** molecular diagnosis, porcine circovirus, spatial transcriptomics, Stereo-seq, tissue microarray (TMA)

## Abstract

Porcine Circovirus type 2 (PCV2) is a key pathogen in pigs that induces host immunosuppression, leading to severe secondary infections such as porcine circovirus-associated disease (PCVAD), resulting in significant economic losses for the swine industry. More recently, it has emerged as a pathogen that needs to be ruled out in porcine xenotransplantation research due to issues related to latent infection. In this study, we established a methodology for distinguishing cell types and detecting the viral transcripts within porcine tissue microarrays using spatial transcriptomics techniques. Random primer capture-based Stereo-seq, followed by unsupervised cell clustering, distinguished four major microstructures within kidney tissue cores, each defined by a distinct marker gene: glomerulus (MAGI2), proximal tubule (CUBN), distal convoluted tubule (SLC8A1), and collecting duct (ERBB4). We further detected PCV2 transcripts in well-segmented proximal tubular cells. The spatial transcriptomics technique used in this study was able to detect both host and microbe transcriptomes with high sensitivity in tissue microarrays re-embedded from archival samples for diagnostic purposes. This suggests that spatial transcriptomics is an advanced pathology technology that integrates spatially resolved gene expression profiling with downstream bioinformatics and computational analysis, enabling the reconstruction of tissue microstructure and the diagnosis of emerging and re-emerging pathogens at the molecular level.

## Introduction

Porcine circovirus type 2 (PCV2) is a small, non-enveloped, single-stranded DNA virus belonging to the genus Circovirus and is the principal causative agent of porcine circovirus-associated disease (PCVAD) affecting systemic organs including respiratory, immune, and urinary system (1, 2). PCV2 infects a wide range of porcine cell types, including monocytes, macrophages, dendritic cells, and endothelial cells, and is characterized by its ability to induce lymphoid depletion, chronic inflammation, and immunosuppression. PCV2 also remains a major concern in xenotransplantation because pigs are the leading donor species for clinical transplantation, and latent or subclinical PCV2 infection may compromise donor organ health or raise concerns about cross-species pathogen transmission, despite the lack of confirmed human infection (3, 4). Due to their importance in the swine and bio-industries, the diagnosis of viral infections largely relies on PCR or NGS techniques, but the analysis of tissue homogenates results in the loss of topological information regarding the lesions or microenvironments.

Transcriptomic approaches have previously been applied to PCV2 infection, though almost exclusively in bulk or dissociated tissue. Bulk RNA-sequencing of lung from genetically divergent pig breeds has identified host responses underlying differential susceptibility to PCV2 disease (5), and whole-transcriptome sequencing of porcine lymph node has profiled mRNA and long non-coding RNA responses to infection (6). At the level of tissue-state inference, an approach based on a compendium of porcine gene expression signatures showed that subclinical PCV2 infection in lymph node is characterized by suppression of a myeloid leukocyte–mediated immune response, in contrast to the inflammatory myeloid activation seen in clinically affected animals (7). More recently, single-cell RNA-sequencing of lung in a PCV2d-infected murine model resolved viral replication and immunosuppressive responses across ten cell types (8).

Spatial transcriptomics is one of the most cutting-edge fields in multi-omics analysis that allows for the identification of tissue microstructure and cell types at the molecular level (9, 10). Recent spatial transcriptomics platforms are capable of detecting not only host RNA but also exogenous RNA including viral transcripts, enabling precise tracking of specific types of infected cells within microstructures (11). This allows for the molecular elucidation of viral pathogenicity by analyzing tissue lesions in conjunction with pathogen-cell interactions. Furthermore, it can be utilized as a reference for transcriptome maps capable of performing antigen screening analysis across systemic organs. One of the most challenging aspects of current spatial transcriptomics is the limitation on the size of tissues that can be analyzed and imaged on a single slide. Consequently, high analysis costs are required when tissue samples are large or numerous. We established a sampling methodology suitable for the simultaneous analysis of tissue structures and virus infection derived from multiple paraffin block samples by applying the tissue microarray (TMA) (12). TMA enables the standardized analysis of multiple tissue samples on a single slide, thereby reducing reagent consumption, experimental variability, and analysis time, although the limited tissue area and potential loss of topological context may restrict the assessment of tissue heterogeneity. Through this, we aimed to delineate the PCV2-infected microstructures and cells from pig renal tissues that had clinical signs but could not be confirmed with latent infection using other molecular biological techniques.

## Methods

### Case selection and differential diagnosis

This study utilized archival samples from routine diagnostic submissions originating from farrow-to-finish herds (approximately 150 sows) with a documented history of PCVAD. PCV2 status was determined at three levels. First, serum was tested for anti-PCV2 antibodies using a commercial ELISA (Porcine Circovirus Type 2 Antibody Test; BioChek B.V., Reeuwijk, the Netherlands), with seropositivity defined as an S/P ratio ≥ 0.5 (13). Second, serum and tissue homogenates were tested for PCV2 genomic DNA by quantitative PCR targeting the capsid-coding region, with a limit of detection of 1.2 × 10^2^ genomic copies/ml (14). Third, tissue sections were examined by immunohistochemistry (IHC) using an anti-PCV2 polyclonal antibody diluted 1:500 in phosphate-buffered saline (15). In total, twenty seropositive pigs aged 7–10 weeks were screened. Of these, ten pigs were excluded because of detectable viremia and/or PCV2 positivity in tissues. The remaining ten pigs were submitted for histopathological examination.

### Tissue microarray

We targeted tissues from pigs suspected of subclinical infection from farms with a prior history of PCVAD outbreaks, where seroconversion was confirmed but viremia was not detected in the serum samples. All submitted animals underwent complete post-mortem examination. All necropsied tissues were fixed in formalin within 2 days, and paraffin-embedded tissues stored for a period of up to 2 years were utilized for TMA construction. Formalin-fixed, paraffin-embedded (FFPE) sections of superficial inguinal lymph nodes, spleen, lung, liver and kidney were stained with hematoxylin and eosin (H&E) and reviewed by a veterinary pathologist (T.O.). Animals showing lesions of PCVAD, including lymphoid depletion and histiocytic or granulomatous replacement of lymphoid follicles, were excluded. Kidneys were additionally screened for necrotizing vasculitis, fibrinonecrotizing glomerulonephritis, and interstitial nephritis (16). In particular, we constructed a TMA using intact kidney tissues confirmed to be PCV2-negative by additional qPCR or IHC as described above. First, differential diagnosis was performed on the prepared tissue slides using H&E staining and IHC, and regions of interest (ROIs) with distinct histological structures were selected. Subsequently, biopsy punches were obtained with core sizes ranging from 1–4 mm suitable for the tissue containing the ROIs at multiple times, and the target core samples were transferred to a new recipient block. The completed TMA blocks were used to perform serial sections for the slide scan areas required by the spatial transcriptomics platform (Figure 1A).

**Figure 1:**
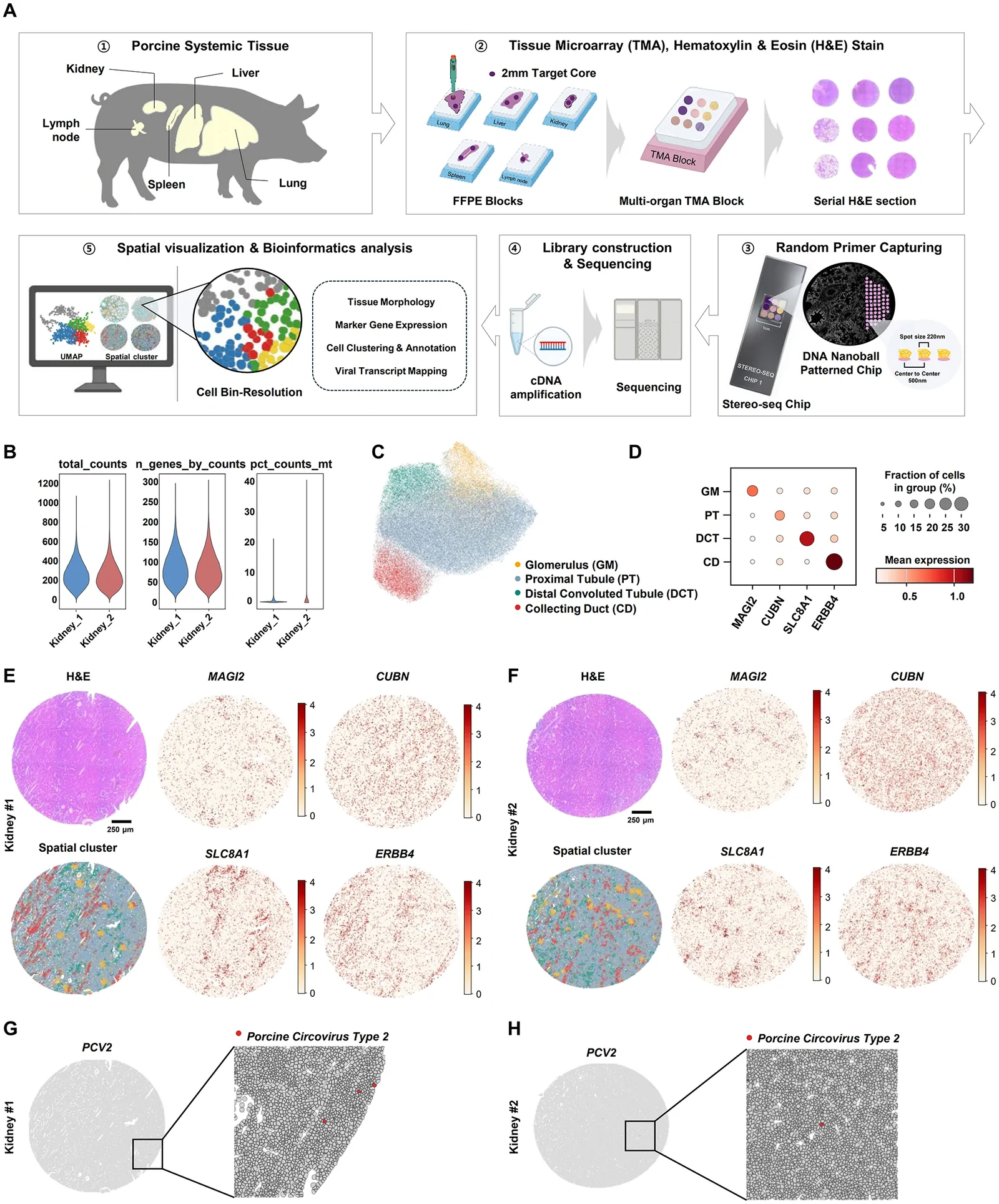
**(A)** Porcine tissue microarray method and stereo-seq workflow. **(B)** Violin plot of the spatial transcriptome data for quality control. **(C)** Uniform Manifold Approximation and Projection (UMAP) representation colored by major cell types. **(D)** Dot plot showing marker gene expression patterns in each cluster. **(E, F)** Hematoxylin and eosin staining and visualization of spatial clusters and marker genes. **(G, H)** Mapping of PCV2 viral transcripts on single cell segmentation.

### Stereo-seq library construction and sequencing

FFPE tissues were sectioned at a thickness of 4μm, and two serial sections were prepared for analysis. The first section was subjected to H&E staining for histological evaluation, while the second was mounted directly onto a Stereo-seq chip N Slide V1.0 (STOmics, Cat. No. 210CN114). Subsequent processing was performed according to the Stereo-seq Transcriptomics Set for FFPE User Manual (Document No. STUM-TT004) using the Stereo-seq Transcriptomics N Kit V1.0 (Cat. No. 211KN114) and Stereo-seq 16 Barcode Library Preparation Kit V1.0 (Cat. No. 111KL160). Briefly, the slides were incubated and subsequently fixed in methanol. Tissue sections were permeabilized under optimized conditions to release RNA molecules, which were captured by DNA nanoball (DNB) arrays on the chip surface. Reverse transcription was then carried out to synthesize cDNA directly *in situ*. Following tissue removal and chip washing, cDNA was amplified by PCR and then quantified using the Qubit dsDNA HS Assay Kit. DNA was enzymatically fragmented and subjected to a second round of PCR to generate sequencing libraries, which were purified with AMPure XP Beads and quantified again prior to sequencing. DNA nanoballs were then prepared using the High-Throughput Sequencing Primer Kit and sequenced on the DNBSEQ-Tx platform, yielding 4.79 billion paired-end reads (Read1: 25 bp, Read2: 59 bp), meeting the manufacturer's recommended sequencing depth of 4 billion reads per 1 cm^2^ Stereo-seq OMNI chip (Complete Genomics/MGI). The generated raw fastq files were processed with the SAW pipeline following the previously described instructions (17).

### Stereo-seq raw data processing

Raw FASTQ data were processed following Stereo-seq Analysis Workflow (SAW) as described in the manufacturer's workflow using SAW (v.8.1.3 for FFPE) (https://github.com/STOmics/SAW). Briefly, during the SAW process, coordinate identifiers (CIDs) and molecular identifiers (MIDs) were extracted from read 1, and the cDNA sequences were extracted from read 2 in the FASTQ files. DNA reads were aligned using STAR against the modified reference porcine genome (assembly version 11.1 + PCV2 constant ORF1). To each gene of each CID, only one read with the same MID was retained for counting. After annotation and count, gene expression matrices with CID were generated. Mapped data were further processed following the Stereopy (v.1.1.22) workflow. Gene expression matrices were loaded from GEF (Gene Expression Format) files at cell bin resolution using the STOmics Stereo (stereo) Python package. Cell boundaries were defined by nucleus image-based cell segmentation, and polygon coordinates for each cell were extracted from the HDF5-formatted GEF file. For genes with duplicated names, only the entry with the highest total expression was retained. The corresponding H&E-stained image of the tissue section was registered to the spatial gene expression matrix using the tissue track lines on the Stereo-seq chip as intrinsic fiducial markers; tissue orientation and the spatial position of the expression data relative to the tissue image were further adjusted manually based on tissue morphology using StereoMap (v.4.2.2) to ensure accurate alignment between the histological image and the corresponding spatial coordinates.

## Results

### Downstream analyses of spatial transcriptome

Kidney TMA sections yielded 39,648 and 50,964 cell bins per tissue biopsy core (Kidney #1 and Kidney #2, respectively; 90,612 cells in total) across 16,278 genes for Stereo-seq analysis. Quality control metrics were computed using Scanpy (v. 1.9.2). For each cell, the total UMI count (total_counts), the number of detected genes (n_genes_by_counts), and the percentage of mitochondrial gene counts (pct_counts_mt) were calculated using sc.pp.calculate_qc_metrics. Mitochondrial genes were defined as ND1, ND2, ND3, ND4, ND4L, ND5, ND6, COX1, COX2, COX3, ATP6, ATP8, and CYTB. QC distributions were visualized with violin plots across samples. Raw count matrices were preserved in the counts layer. Total counts per cell were normalized to 10,000 (sc.pp.normalize_total, target_sum = 1 × 104), followed by log1p transformation (sc.pp.log1p). Highly variable genes (HVGs) were identified using sc.pp.highly_variable_genes with default parameters (no restriction on the number of top genes). Expression values were then scaled with a maximum value of 10 (sc.pp.scale, max_value = 10) (Figure 1B).

Principal component analysis (PCA) was performed using sc.tl.pca. Based on variance ratio inspection, the top 10 principal components (n_pcs = 10) were selected for neighborhood graph construction (sc.pp.neighbors). Uniform Manifold Approximation and Projection (UMAP) was applied for two-dimensional visualization (sc.tl.umap). Unsupervised clustering was performed using the Leiden algorithm (sc.tl.leiden, resolution = 0.5). For combined analysis of Kidney #1 and Kidney #2, the two samples were concatenated using raw count matrices using sc.concat. The merged dataset was preprocessed as described above with n_pcs = 15. Batch effects between TMA regions were corrected using Harmony integration (sc.external.pp.harmony_integrate, key = ‘TMA'). The corrected embedding (X_pca_harmony) was used for neighborhood graph construction (n_pcs = 15), UMAP computation, and Leiden clustering (resolution = 0.5).

### Visualization of spatial cluster

To incorporate spatial context, a spatial-aware Leiden clustering approach was applied. For each cell, the 10 nearest spatial neighbors were identified using scikit-learn's NearestNeighbors based on spatial coordinates. PCA embeddings of neighboring cells were concatenated to create a spatially smoothed representation, which was then reduced to 20 principal components using scikit-learn's PCA. A neighborhood graph was constructed from this smoothed embedding, and Leiden clustering was performed at a resolution of 1.5. Spatial Leiden clusters were further condensed into four major renal compartments—proximal tubule (PT; CUBN), distal convoluted tubule (DCT; SLC8A1), collecting duct (CD; ERBB4), and glomerulus (GM; MAGI2)—based on marker gene expression patterns. Differentially expressed genes (DEGs) were identified using the Wilcoxon rank-sum test (sc.tl.rank_genes_groups, method = ‘wilcoxon') across Leiden clusters. Marker gene expression was visualized using dot plots (sc.pl.dotplot) and a custom marker analysis module (sceleto2.markers.marker) (Figures 1C, D). Simultaneously, the spatial annotation of each cell cluster was corroborated by H&E staining, mapping expression locations of the corresponding canonical marker genes to histological microstructures within the kidney tissue at single-cell polygon resolution (Figures 1E, F).

### Detection of PCV2 infection

PCV2-positive cells were identified based on the expression of PCV2_ORF1. Cells with nonzero PCV2_ORF1 counts were classified as PCV2-positive. The spatial distribution of PCV2-positive cells was visualized using cell polygon plots with zoomed-in views around each positive cell. Spatial gene expression and cell annotations were visualized using a custom cell polygon plotting function that renders individual cell boundaries as filled polygons on tissue coordinates. We mapped the viral sequences of PCV2 genotypes using a conserved PCV2_ORF1 sequence to the renal spatial transcriptome data and specifically detected PCV2d (MN545963.1) transcripts (PCV2_ORF1) in a total of 8 PCV2-positive cells across both kidney cores. In Kidney #1, all four PCV2-positive cells were localized within the proximal tubule (PT) regions; in Kidney #2, PCV2-positive cells were distributed across the glomerulus (*n* = 1), distal convoluted tubules (*n* = 2), and proximal tubule (*n* = 1). PCV2-positive cells were highlighted in red at single-cell-bin resolution (Figures 1G, H).

## Discussion

Current spatial transcriptomics technologies can be broadly divided into two main groups. Sequencing-based approaches enable near whole-transcriptome profiling but generally provide modest spatial resolution (e.g., Visium: 2,000 nm and Stereo-seq: 500 nm). Imaging-based platforms achieve single-cell to subcellular resolution (e.g., Xenium: 50 nm and MERFISH: 100 nm), although they are typically limited to predefined panels of target genes. The Stereo-seq we utilized in this study is a cutting-edge transcriptomics technology that dramatically increases spatial resolution by reducing the DNA nanoball array spot spacing to less than 500 nm. Cluster annotation of segmented cells in Stereo-seq enabled a clear delineation of the renal structure from a histologic perspective. The kidney can be broadly divided into the cortex, outer medulla, and inner medulla; among these, the cortex is a heterogeneous region containing a nephron composed of a renal corpuscle and tubule, as well as a collecting duct, making it suitable for spatial transcriptomic analysis (18). Consequently, the distribution of four clusters classified according to similar transcript expression patterns allowed for the visualization of the kidney in a manner consistent with the histological classification structure of the cortex. This also aligns with the expression patterns of known canonical markers in the cells of each structure in the perspective of molecular biology (19). Further deep clustering is required to more clearly define the novel microstructures at the molecular level.

Another advantage of Stereo-seq is that it is species-agnostic due to the use of random primers. Therefore, it can detect not only the host transcriptome but also microbial transcripts; we were able to detect PCV2 with high sensitivity in porcine kidney TMA tissues from asymptomatically infected animals that had not been diagnosed by quantitative PCR. This likely reflects the limited sensitivity of conventional assays for focal, low-copy infection: qPCR of bulk homogenates dilutes signal arising from few infected cells, and IHC requires antigen to accumulate above the threshold for detection. Spatially resolved transcriptomics retains this signal *in situ* at sub-cellular resolution, indicating that conventional negativity does not exclude low-level PCV2 infection. PCV2 is the causative agent of PCVAD and causes wasting disease due to co-infection with other pathogens caused by immunosuppression; however, when infected alone, it is often subclinical and frequently excluded from early diagnosis (20). Also, PCV2 frequently contaminates biological products, including cell lines and vaccines, and has been highlighted in the context of xenotransplantation (3), ensuring pathogen-free donor pigs is a central biosafety requirement. Regulatory frameworks and expert consensus emphasize rigorous screening for porcine viruses, including PCV2, because immunosuppressed transplant recipients may be particularly vulnerable to zoonotic agents or indirect graft dysfunction caused by donor-derived infections (3, 4). In this context, a highly sensitive microbial transcript detection method utilizing spatial transcriptomics holds value as a next-generation diagnostic platform.

Kidney was selected as the target organ for three reasons. First, PCV2 nucleic acid has been demonstrated in renal tubular epithelial cells by *in-situ* hybridization in naturally infected pigs, establishing the renal tubule as a documented site of viral localization (21). Second, the renal cortex has a stereotyped microstructure—renal corpuscle, proximal and distal tubule, and collecting duct—which serves as a histological reference for validating unsupervised spatial clusters. Third, the kidney is a target organ in PCVAD and is routinely archived in diagnostic submissions, making it directly relevant to the archival-block workflow proposed here. Lymphoid tissues, although the principal site of PCV2 replication, were excluded, as their histological homogeneity following lymphoid depletion offers a poor substrate for validating microstructure-level resolution. The animals were seropositive and non-viremic at collection, a profile consistent with a post-viremic phase of infection. Defining the temporal distribution of viral transcripts across systemic organs would require prospective, serially sampled cohorts. In addition, given that viral transcripts were detected in different cell types within the tissues, additional confirmation using conventional *in-situ* hybridization would provide further validation and improve the sensitivity and specificity of subclinical infection detection.

## Conclusion

This study utilized an advanced spatial transcriptomics platform based on random primer capturing to reconstruct the complex structure of the porcine renal cortex at the molecular level and detected subclinical PCV2 infection in its microstructures. These results suggest that spatial transcriptomics enables highly sensitive virome analysis within the context of tissue architecture and propose that it represents an important research field capable of simultaneously enhancing the economic viability of the swine industry and the stability of the future biotechnology.

## Data Availability

Processed spatial data analyzed in this study are available in the Zenodo repository (https://doi.org/10.5281/zenodo.20340406). Raw data are available from the corresponding author upon request. The custom code used for data analysis is available in the Zenodo repository (https://doi.org/10.5281/zenodo.20340406).
